# Increase in Diarrheal Disease Associated with Arsenic Mitigation in Bangladesh

**DOI:** 10.1371/journal.pone.0029593

**Published:** 2011-12-28

**Authors:** Jianyong Wu, Alexander van Geen, Kazi Matin Ahmed, Yasuyuki Akita Jahangir Alam, Patricia J. Culligan, Veronica Escamilla, John Feighery, Andrew S. Ferguson, Peter Knappett, Brian J. Mailloux, Larry D. McKay, Marc L. Serre, P. Kim Streatfield, Mohammad Yunus, Michael Emch

**Affiliations:** 1 Department of Environmental Sciences and Engineering, University of North Carolina at Chapel Hill, Chapel Hill, North Carolina, United States of America; 2 Lamont-Doherty Earth Observatory of Columbia University, Palisades, New York, United States of America; 3 Department of Geology, University of Dhaka, Dhaka, Bangladesh; 4 Department of Civil Engineering and Engineering Mechanics, Columbia University, New York, New York, United States of America; 5 Department of Geography, University of North Carolina at Chapel Hill, Chapel Hill, North Carolina, United States of America; 6 Department of Earth and Environmental Engineering, Columbia University, New York, New York, United States of America; 7 Department of Earth and Planetary Sciences, University of Tennessee, Knoxville, Tennessee, United States of America; 8 Department of Environmental Sciences, Barnard College, New York, New York, United States of America; 9 International Centre for Diarrhoeal Disease Research, Dhaka, Bangladesh; 10 Carolina Population Center, University of North Carolina at Chapel Hill, Chapel Hill, North Carolina, United States of America; Aga Khan University, Pakistan

## Abstract

**Background:**

Millions of households throughout Bangladesh have been exposed to high levels of arsenic (As) causing various deadly diseases by drinking groundwater from shallow tubewells for the past 30 years. Well testing has been the most effective form of mitigation because it has induced massive switching from tubewells that are high (>50 µg/L) in As to neighboring wells that are low in As. A recent study has shown, however, that shallow low-As wells are more likely to be contaminated with the fecal indicator *E. coli* than shallow high-As wells, suggesting that well switching might lead to an increase in diarrheal disease.

**Methods:**

Approximately 60,000 episodes of childhood diarrhea were collected monthly by community health workers between 2000 and 2006 in 142 villages of Matlab, Bangladesh. In this cross-sectional study, associations between childhood diarrhea and As levels in tubewell water were evaluated using logistic regression models.

**Results:**

Adjusting for wealth, population density, and flood control by multivariate logistic regression, the model indicates an 11% (95% confidence intervals (CIs) of 4–19%) increase in the likelihood of diarrhea in children drinking from shallow wells with 10–50 µg/L As compared to shallow wells with >50 µg/L As. The same model indicates a 26% (95%CI: 9–42%) increase in diarrhea for children drinking from shallow wells with ≤10 µg/L As compared to shallow wells with >50 µg/L As.

**Conclusion:**

Children drinking water from shallow low As wells had a higher prevalence of diarrhea than children drinking water from high As wells. This suggests that the health benefits of reducing As exposure may to some extent be countered by an increase in childhood diarrhea.

## Introduction

Diarrheal diseases and arsenic (As) poisoning are both severe health problems that are related to the quality of drinking water in Bangladesh [1], [2], [3]. High population density, frequent flooding, and poor sanitation render surface water bodies in this country particularly vulnerable to fecal contamination, thus leading to a high prevalence of diarrheal diseases, especially among children under five [4]. In the 1970s, the infant mortality rate attributable to diarrhea was up to 36 per 1000 live births and accounted for a significant proportion of total infant mortality (140/1000) [1]. Although diarrhea is still one of the leading causes of childhood mortality, the number of deaths attributed to diarrhea has decreased markedly down to 13 per 1000 infants out of a total infant mortality of 65 per 1000 live births [5]. It is often assumed that the installation of millions of tubewells that now provide drinking water to 95% of the rural population in Bangladesh contributed to the decline in childhood mortality because the likelihood of microbial contamination of groundwater is much lower than that of surface water. This assumption is not supported by contemporary analysis [6], [7], however, and the reduction in childhood mortality probably reflects multiple interventions including advances in treatment, widely administered oral rehydration salts in particular, as well as improvements in water supply, sanitation, personal hygiene, and nutrition [8], [9], [10], [11].

A new health problem related to the quality of drinking water was discovered when high levels of As were detected in many shallow tubewells in the 1990s. Until mitigation started in earnest a decade ago, about a quarter of the population of Bangladesh was exposed to As levels above the national standard for drinking water of 50 µg/L, whereas half the population was exposed above the WHO guideline for drinking water of 10 µg/L [12]. Significant health impacts from drinking high-As groundwater have since been reported including an increase in all-cause mortality due in considerable to cardiovascular disease [13], skin lesions [14], various forms of cancer [3], and reduced intellectual function in exposed children [15].

The most effective forms of As mitigation have relied on providing alternative groundwater sources to reduce exposure rather than attempts to remove As from groundwater, collect and store rainwater, or treat surface water [16]. This is because the spatial distribution of As in groundwater is highly variable and shallow tubewells are relatively inexpensive (<$150) to install, convenient to use, and require little maintenance. Blanket testing of tubewells with field kits throughout the affected regions of Bangladesh in 2000-05 is estimated to have induced 30-50% of households with a shallow (typically <140 ft deep) high-As well to switch their consumption to a neighbor's well that is low in As, and often also shallow [16], [17], [18], [19]. The next most common form of mitigation has been the installation of tens of thousands of more expensive deep (≥300 ft) community wells by the government and non-governmental organizations in villages throughout the country with a particularly high proportion of high-As wells [16], [20], [21].

The possibility that some forms of As mitigation such as pond water treatment with a sand filter, rainwater harvesting, and dug wells open to surface contamination could increase the burden of diarrheal disease has been raised [22], [23]. Less attention has been paid, however, to the possibility that some categories of wells that are low in As could be particularly vulnerable to microbial contamination. The present analysis of a unique set of diarrheal disease data from Bangladesh is motivated by hydrogeological considerations suggesting that shallow tubewells that are low in As might be particularly prone to microbial contamination [24]. Monthly monitoring of 125 shallow tubewells in two separate regions of Bangladesh has since confirmed that groundwater pumped from shallow low-As wells is more likely to contain the fecal indicator *E. coli* than groundwater from shallow wells that are high in As [25]. This is probably because shallow low-As aquifers are less effectively protected from shallow contaminated sources of recharge by a surface layer of fine-grained sediment often associated with shallow high-As aquifers [26]. The concern is, therefore, that the growing share of the population in Bangladesh that is switching to low-As wells, many of which are likely to be shallow, could be exposed to higher levels of fecal contamination and diarrheal disease pathogens. This cross-sectional study tests this hypothesis by determining whether children drinking from low-As wells are indeed more likely to suffer more diarrheal disease.

## Materials and Methods

### Ethics statement

The study was approved by the institutional review boards of ICDDR, B, Columbia University and the University of North Carolina, Chapel Hill. A verbal consent was obtained from each participant by ICDDR,B community health research workers. Written consent was not obtained because many participants are illiterate and the data were analyzed anonymously.

### Study area and design

**Figure 1:**
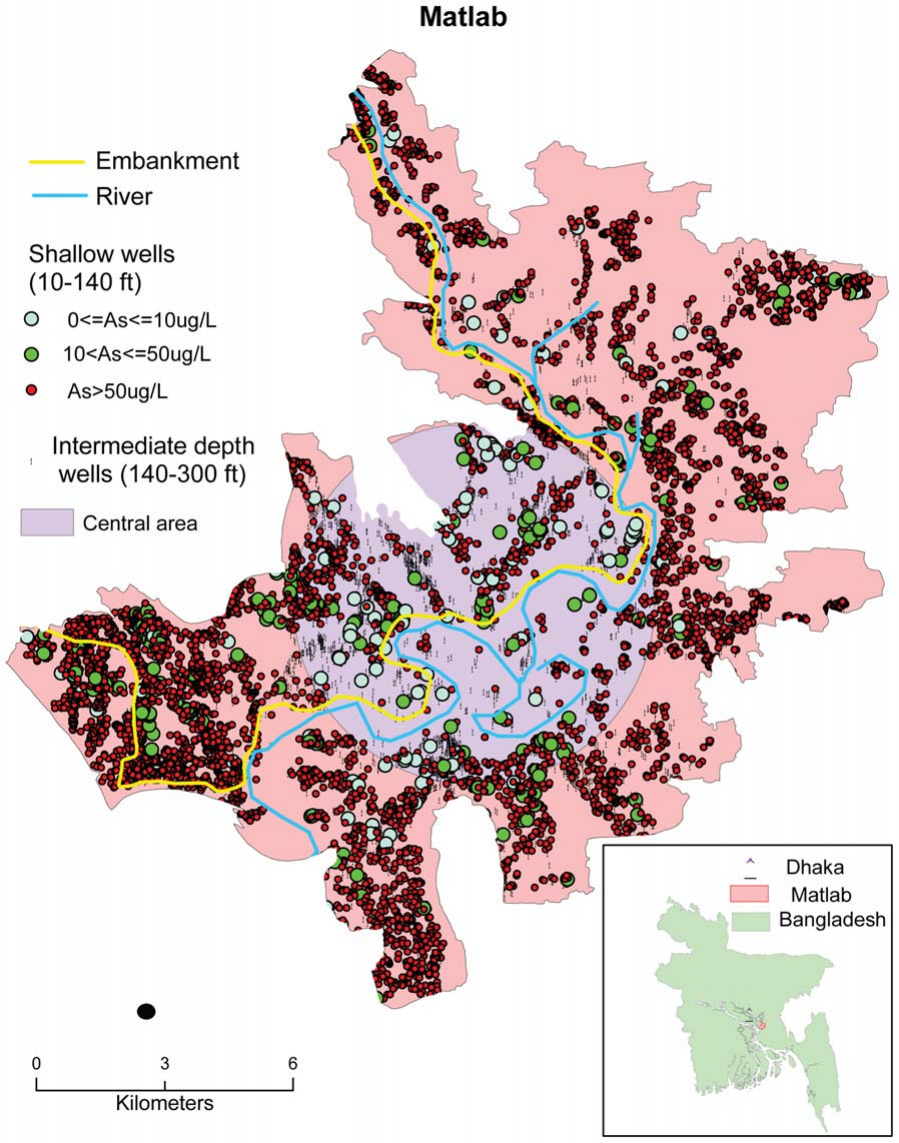
Map of the study area of Matlab, Bangladesh showing the spatial distribution and As content of tubewells for different depth intervals.

The data for this study were collected in Matlab, Bangladesh, which is located approximately 50 km southeast of Dhaka (Figure 1). Like other rural areas in Bangladesh, the main economic activities in Matlab are agriculture and fishing. Our analysis of potential relationships between tubewell characteristics and childhood diarrhea was applied to all 142 villages of Matlab.

A cross-sectional study was designed to explore the relationship between childhood diarrhea and As levels in groundwater. The outcome is the average daily prevalence of diarrhea in a *bari*, a patrilineally-related cluster of households. The measure of exposure is the As level measured in tubewell water. Associations between outcome and exposure were analyzed using logistic regression models.

### Data collection and management

An extensive Health and Demographic Surveillance System (HDSS) covering the entire study area includes monthly visits to each household to collect population and health data. The definition of diarrhea in this study follows the one proposed by UNICEF/WHO [27] of 3 or more bloody or watery stools during the previous 24 hours. The location and total population of each of the 10,945 *baris* in Matlab are known from HDSS records. Based on the birth date of each person, we calculated the number of children under five in each *bari*. The population within 100 meters around each *bari* was calculated as a local measure of population density.

A wealth index for each household was calculated using asset information collected during censuses conducted by ICDDR, B in 1996 and 2005. The wealth index was derived from principal component analysis of a series of ownership variables that includes a bed, bicycle, blanket, lamp, watch and one ordinal variable, the material used to construct the household's dwelling [28]. The wealth index is the first principal component of this analysis and accounts for 31% of the variance. The wealth index does not include a measure of household education, although the latter is likely to be related to household wealth. A *bari*-level wealth index was calculated as the mean wealth for all of the households in a *bari* [28]. *Baris* were sorted from lowest to highest wealth index and then divided into five groups. A higher score indicates a higher wealth index. The adult female education (age≥ 15 years) in Matlab was surveyed in 2005. Based on this dataset, a *bari*-level education index was created by calculating the average year of adult female education in a *bari*, then the index was classified into 5 categories: 1) less than 1 year, 2) 1 year to less than 3 years, 3) 3 year to less than 5 years, 4) 5 years to less than 7 years and 5) 7 years and above. A flood-control embankment built in 1990 bisects Matlab into a protected area to the west side and an unprotected area to the east (Figure 1). About one third of the *baris* in Matlab are located within the flood control area whereas the others are unprotected.

The location and depth of 10,869 tubewells in Matlab was recorded when water samples were collected for As testing in 2002–2003. Concentrations of As in tubewell water were measured using hydride generation atomic absorption spectrometry (HG-AAS) as well as field-kits [29]. Previous studies have shown that As concentrations in groundwater generally do not vary over time in Bangladesh, although there are exceptions especially at shallow depths [30]. In response to well testing, a considerable fraction of the population likely switched away from high-As tubewells to nearby existing low-As wells after test results were communicated. Several hundred new deep tubewells were also installed throughout Matlab as community sources of low-As water in 2005. For lack of more specific information, people living in *baris* are assumed to drink from the same well throughout the 2000-06 study period. However, individual years are also considered in the regressions.

Well depth was recorded by asking the most informed person in the household how many PVC pipes of standard 20-ft length were used to construct the well. Interviewers calculated the depth of a well from the number of PVC pipes and the typical 10-ft length of the filter at the bottom. The actual depth of household wells has only occasionally been measured and compared to reported depths but not, to our knowledge, systematically and on any large scale. Patterns reported at the village-scale between the depth of wells, the local geology, and their As content provide one indication that depths are, by-and-large, correctly recalled and reported. In the case of deep tubewells (depths >300 ft), the cost of installation is typically not born by local households but by NGOs or the government's Department of Public Health Engineering (DPHE). There are credible reports that contractors or local officials in charge of these community wells install them at a depth shallower than reported, but this does not affect the present analysis which is focused on the much larger number of shallow and intermediate-depth wells.

A geographic information system (GIS) was used to integrate the available data. A total of ∼7,000 *baris* with children under five were linked to tubewells using their identification number. In brief, each *bari* has a unique identification number, and a tubewell was labeled with the identification number of a *bari* where the people who drink from it live. Among these *baris*, ∼2,800 could unambiguously be assigned a single tubewell. An additional ∼2,400 *baris* had two or more choices of tubewells and the remaining ∼1,900 could not be assigned a tubewell using their identification number. For *baris* with more than one tubewell or without an assigned tubewell, the nearest tubewell in the database was chosen as the most likely water source for that *bari*. The subset of *baris* for which tubewells could be assigned unambiguously were also considered in a separate regression.

### Statistical analysis

The dependent variable of this analysis is childhood diarrhea. To create a binary dependent variable, the average daily prevalence in a year was calculated using the average daily diarrhea cases (the average daily diarrhea cases = total diarrhea cases in a year divided by 12) divided by the total number of children under five. We excluded *baris* that had no children under five. The daily prevalence of each *bari* was then compared with the average daily prevalence. The outcome was coded as 1 if the daily prevalence of each *bari* was larger than the average and as 0 otherwise.

**Figure 2:**
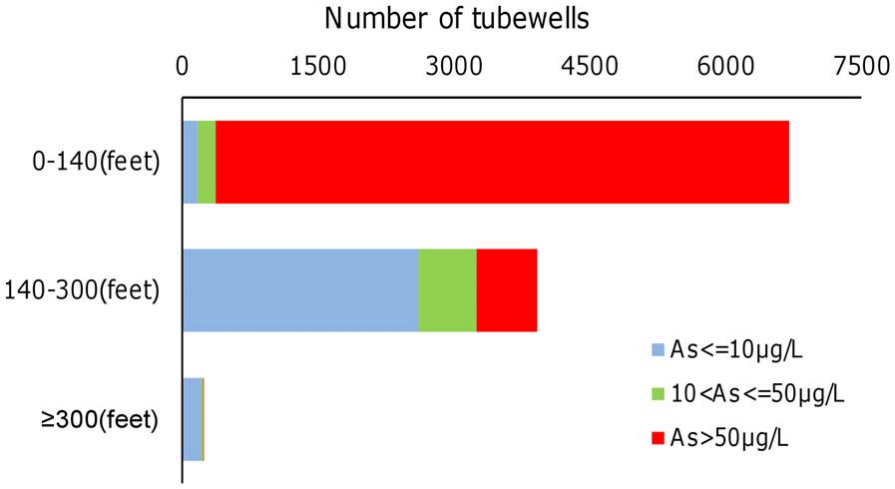
Total number of tubewells in Matlab for different depth intervals and As concentration ranges.

Both univariate and multivariate analyses were conducted. For univariate analyses, the As concentration of the assigned tubewell is the only independent variable and was reconstructed as a categorical variable relative to the WHO guideline for As in drinking water of 10 µg/L and the Bangladesh standard of 50 µg/L, the latter of which is also the As threshold that was used to label millions of tubewells in Bangladesh as safe or unsafe [16]. The three ranges of tubewell As concentrations were defined as very low (≤10 µg/L), low (10<As≤50 µg/L), and high (As>50 µg/L) in both the univariate and the multivariate regression models. Depth, location (central vs. peripheral area in Matlab), population density, wealth, education and flood control were used as control variables in the univariate analyses. Well depth was classified into three categories: shallow (10<depth<140 ft), intermediate (140≤ depth<300 ft) and deep (≥300 ft). The groupings reflect natural breaks in the depth distribution of wells in Matlab (Figure S1). Only the shallow and intermediate depth intervals were considered in this analysis because the number of deep wells is small and groundwater from the vast majority of these wells (97%) contains no more than 50 µg/L As (Figure 2). In separate analyses reflecting the greater concentration of shallow wells that were low or very low in As in the central of Matlab, the region was divided into a central and a peripheral area (Figure 1). Population density was classified in three groups of comparable size: low (31–1000 per km^2^); intermediate (1000–3000 per km^2^) and high (≥3000 per km^2^). The flood control variable is binary and distinguishes *baris* that are protected by the embankment from those that are not. For the multivariate analyses, the independent variables include As level, population density, wealth index and flood control, whereas tubewell depth is used as a control variable, using the same categories of variables as for the univariate analysis. Education index was not included in the multivariate analysis because it was strongly correlated with wealth index (Spearman rank correlation coefficient r = 0.51, p<0.0001, and n = 49475), which was included the models.

Odds ratios (ORs) and their 95% confidence intervals (CIs) were calculated based on the logistic regression models and used to examine the association between diarrhea and risk factors. The “odds” are defined as the probability of an outcome event (for example, a diarrhea case) occurring divided by the probability of the event not occurring. In general, the odds ratio for a predictor of an independent variable is defined as the degree to which the odds of the outcome increase (OR>1.00) or decrease (OR<1.00) when the value of the independent variable increases by 1 unit. Here, the OR corresponds to a 1 unit change in categorical risk factors including tubewell As (3 categories), population density (3 categories), wealth index (5 categories), or flood control (2 categories). A positive or negative association is indicated if the OR is significantly (p<0.05) larger or smaller than 1, respectively. All statistical analyses were conducted using SAS 9.2 (SAS Inc., Cary, NC).

## Results

### Description of the datasets

The childhood diarrhea cases, the number of children under five, as well as population data, were collected from 2000 to 2006 in 142 villages of Matlab, Bangladesh. Diarrhea cases were aggregated to the *bari*-level, which are the unit of analysis for this study. The number of *baris*, with children under five in Matlab remained relatively steady at ∼64% of all *baris* over the 7 year study period (Table 1). The average number of children per *bari* within this group ranged from 3.7 to 4.5. The average daily prevalence during the whole study period was 25 diarrhea cases per 1000 children under five. The average daily prevalence varied each year with the highest rate (35 cases/1000 children under five) in 2003 and the lowest rate in 2006 (14 cases/per 1000 children under five). On average, there were 3 episodes of diarrhea per *bari* per month for an average of 4 children under five per *bari* (*baris* having no children under five were excluded from the calculation).

**Table 1:** Population and childhood diarrheal disease in 142 Matlab villages.

Year	No. *baris* having children	No. children under five	Total No. diarrhea Cases	Average daily diarrhea cases	Average daily prevalence
2000	7066	31586	10091	841	0.027
2001	7240	31776	8915	743	0.023
2002	7369	31808	11248	937	0.029
2003	7435	31077	12891	1074	0.035
2004	7494	30099	9776	815	0.027
2005	7445	28537	6279	523	0.018
2006	7357	26958	4610	384	0.014
Average	7344	30263	9116	760	0.025

Note: Average daily diarrhea cases =  total number of diarrhea cases/12 based on the diarrhea were collected by 12 visits (days) each year.

Average daily prevalence =  Average daily diarrhea cases/ the number of children under five.

In Matlab, there are approximately 12,000 tubewells which provide drinking water to more than 95% of the residents [29] (Figure 1). Among those wells, the As content of shallow (<140 ft) tubewells is overwhelmingly high, with only 372 (5.5%) out of 6705 wells in that depth range containing ≤50 µg/L As (Figure 2). In contrast, 3248 (83%) out of 3922 intermediate-depth wells (140-300 ft) contain ≤50 µg/L As. Shallow wells that are low or very low in As are concentrated in the central portion of Matlab, although 125 out of 142 villages in Matlab include at least one shallow well with ≤50 µg/L As (Figure 1). Among tubewells which were used by *baris* with children, the number of shallow wells is nearly 2 times higher than the number of intermediate-depth wells (Table 2).

**Table 2:** Number of shallow and intermediate depth tubewells in *baris* with one or more children.

	10-140ft	140-300 ft
As≤10 µg/L	116	1510
10<As≤50 µg/L	123	407
As>50 µg/L	3920	439

### Association between childhood diarrhea and As and other risk factors

Univariate logistic regressions show that the rate of childhood diarrhea decreases significantly with an increase in As levels (As was classified into 3 categories or levels, a higher As level corresponds to higher As content) regardless of flood protection (95% CIs<1.00, p<0.001) or population density (95% CIs<1.00, p<0.001) (Table 3). In both the central and the peripheral areas, As level is negatively associated with childhood diarrhea. However, the association is significant only in the peripheral area (OR = 0.92, 95% CI: 0.90–0.95). The rate of childhood diarrhea also decreases when As increases in the middle three of the five wealth index categories as well as in the last four education index categories. The inverse association between childhood diarrhea and tubewell As is statistically significant (p<0.05) for individual years, with the exception of 2004. There is a marked difference when the two depth intervals are considered separately, however. The inverse relationship between childhood diarrhea and well As holds for shallow wells (OR = 0.92, p = 0.009), but not for intermediate-depth wells.

**Table 3:** Associations between childhood diarrhea and tubewell arsenic in Matlab from 2000 to 2006 derived by univariate logistic regressions.

Control variables	n	p	OR	95%CI
Depth (ft)				
10–140	29111	0.009	0.92	0.86–0.98
140–300	16489	0.594	0.99	0.95–1.03
Flood control				
Yes	16863	<0.001	0.83	0.80–0.87
No	28737	<0.001	0.94	0.91–0.96
Location				
Central	15211	0.491	0.99	0.95–1.02
Peripheral	34951	<0.001	0.92	0.90–0.95
Population density				
0–1000	13157	<0.001	0.89	0.85–0.93
1000–3000	15191	<0.001	0.92	0.88–0.95
≥3000	17252	<0.001	0.90	0.87–0.93
Wealth index				
1 (poorest)	2510	0.063	0.91	0.82–1.01
2	11991	<0.001	0.91	0.87–0.95
3	19605	<0.001	0.90	0.87–0.93
4	9172	<0.001	0.86	0.82–0.91
5 (richest)	1697	0.096	0.90	0.79–1.02
Education				
<1 year	3708	0.338	0.96	0.88–1.05
1–3 years	12320	<0.001	0.92	0.88–0.97
3–5 years	17905	<0.001	0.88	0.85–0.91
5–7 years	11063	<0.001	0.92	0.88–0.96
≥ 7 years	5166	0.042	0.94	0.88–1.00
Year				
2000	6279	<0.001	0.87	0.82–0.93
2001	6436	<0.001	0.89	0.84–0.95
2002	6540	0.001	0.91	0.86–0.96
2003	6597	0.011	0.93	0.88–0.98
2004	6641	0.071	0.95	0.90–1.01
2005	6590	<0.001	0.88	0.83–0.93
2006	6517	<0.001	0.85	0.80–0.91

As was classified into 3 levels: 1: very low As (≤10 µg/L), 2: low As (10–50 µg/L) and 3: high As(>50 µg/L). The very low As group was taken as the baseline group in the comparison and the odds ratio reflects the change of diarrhea risk when the arsenic level increases one unit.

Multivariate regression models that consider various risk factors simultaneously are consistent with the univariate results (Table 4). For shallow wells, the model continues to indicate a decrease in childhood diarrhea associated with an increase in well As (OR = 0.89, p = 0.001). Children drinking water from low-As (10–50 µg/L) tubewells are 1.11 times (1.11 = 1/OR) more likely to have diarrhea than children drinking from high-As (>50 µg/L) tubewells. Children drinking from very low-As (≤10 µg/L) tubewells are 1.26 times (1.26 = 1/OR^2^) more likely to have diarrhea than children drinking from high-As (>50 µg/L) tubewells (Table 4). The same model shows a significant increase in childhood diarrhea with increasing population density (OR = 1.15, p<0.001) and, conversely, a decrease in diarrhea with increasing wealth index (OR = 0.90, p<0.001). There also is a significant protective effect against childhood diarrhea from flood control (OR = 0.72, p<0.001). When the multivariate models were adjusted by year, the associations between diarrhea and As, flood control, population density and wealth index were not changed. The negative association between diarrhea and the year (OR = 0.89, 95% CI: 0.88-0.90) simply shows that childhood diarrhea cases decreased during the study period. For the intermediate-depth wells, the multivariate models show the same direction and magnitude for the effects of population density, wealth index, flood control, and year on childhood diarrhea. However, the relationship between diarrhea and well As is not significant in the intermediate depth interval (OR = 0.99, p = 0.642) (Table 4).

**Table 4:** Multivariate analysis of associations between childhood diarrhea and related factors.

Control variables	Independent variables	n	p	OR	95%CI
Unstratified	As	49475	<0.001	0.92	0.90–0.94
	Flood control		<0.001	0.79	0.76–0.82
	Population density		<0.001	1.14	1.12–1.17
	wealth index		<0.001	0.87	0.85–0.89
10<depth<140 ft	As	28654	0.001	0.89	0.84–0.96
	Flood control		<0.001	0.72	0.68–0.76
	Population density		<0.001	1.15	1.11–1.18
	wealth index		<0.001	0.90	0.87–0.92
140≤depth<300ft	As	16321	0.642	0.99	0.95–1.03
	Flood control		<0.001	0.89	0.83–0.95
	Population density		<0.001	1.15	1.10–1.19
	wealth index		<0.001	0.87	0.84–0.90
Unstratified	As	49475	<0.001	0.92	0.90–0.94
	Flood control		<0.001	0.79	0.76–0.82
	Population density		<0.001	1.17	1.14–1.19
	wealth index		<0.001	0.87	0.85–0.89
	Year		<0.001	0.90	0.89–0.91
10<depth<140 ft	As	28654	0.001	0.89	0.84–0.96
	Flood control		<0.001	0.72	0.68–0.76
	Population density		<0.001	1.17	1.14–1.21
	wealth index		<0.001	0.90	0.87–0.92
	Year		<0.001	0.89	0.88–0.90
140≤depth<300ft	As	16321	0.591	0.99	0.95–1.03
	Flood control		<0.001	0.89	0.83–0.95
	Population density		<0.001	1.17	1.13–1.22
	wealth index		<0.001	0.87	0.83–0.90
	Year		<0.001	0.90	0.88–0.91

As was classified into 3 levels: 1: very low As (≤10 µg/L), 2: low As (10–50 µg/L) and 3: high As(>50 µg/L). The very low As group was taken as the baseline group in the comparison.

In univariate regressions, the inverse relationship between childhood diarrhea holds for watery but not for bloody diarrhea (Table S1). The inverse relationship also holds for each of the three main seasons in Bangladesh. However, childhood diarrhea and well As are inversely related only for children more than 2 years old. When the population density of children under 5 and tubewell density are considered in multivariate logistic models, childhood diarrhea is still significantly associated with arsenic levels in shallow wells (OR = 0.89, 95% CI: 0.83–0.95) but not in intermediate-depth wells (OR = 0.98, 95%CI: 0.94–1.02) (Table S2). For both shallow and again intermediate-depth wells, childhood diarrhea is positively associated with the population density of children but negatively associated with tubewell density. The associations between childhood diarrhea and flood control and wealth do not change when the two depth intervals are considered separately (Table S2).

## Discussion

The quantity of diarrheal disease data systematically collected in Matlab over a period of seven years is to our knowledge unprecedented for a population that drinks primarily untreated groundwater. The available information provides a unique perspective on potential associations between diarrheal disease and tubewell characteristics and therefore, indirectly, groundwater quality.

The diarrheal disease data were categorized according to the depth and As content of the tubewell that a child is most likely to drink from because of independent evidence that shallow low-As wells are likely to be particularly vulnerable to microbial contamination from concentrated shallow sources such as latrines and ponds. This prediction, based on recent data for the fecal indicator *E. coli* obtained in the context of As-related studies in Bangladesh [24], [25] is here borne out at the population level by the analysis of actual diarrheal disease patterns.

A shallow source of microbial contamination is consistent with the increase in diarrheal disease in proportion to population density [25], even if factors above ground that do not involve groundwater could also drive such a relationship. The dependence of childhood diarrhea on wealth builds confidence in the analysis because it is well established that social-economic status and education, approximated here by the wealth index, impact the likelihood of many infectious diseases [28], [31]. Children living in poor families often do not have access to clean water and their hygiene is not as good. Children from poor families are also more likely to suffer from malnutrition [32]. The lack of a significant relationship between well As and diarrhea for children up to two years old might be an independent indication that well water is a significant source of diarrheal disease because younger children are less likely to drink tubewell water. The significant reduction in diarrheal disease associated with flood control has, to our knowledge, not been demonstrated quantitatively before. The new finding is consistent with the association of elevated *E. coli* and thermotolerant coliforms in tubewell water within a flood-prone area of Matlab [33].

The multivariate model that controls for other risk factors indicates that, on average, children under five who drink untreated groundwater from a shallow well with 10-50 µg/L As are 11% more likely to suffer from diarrheal disease relative to children drinking from a well with >50 µg/L As (Table 4). This is the As threshold that was used to label millions of tubewells throughout the country and that a significant proportion of households responded to by switching to a nearby well [16]. Relative to the WHO guideline, the difference is even more striking with childhood diarrhea 26% more likely for children drinking from a shallow well with ≤10 µg/L compared to wells with >50 µg/L. This is consistent with the highest difference in frequency of *E. coli* detection previously reported for shallow wells meeting the WHO guideline for As compared to wells with >50 µg/L [25].

The mechanism underlying the inverse relationship between diarrhea and As in groundwater is probably related to local variations in groundwater recharge. While most microbial pathogens contained in surface water are retained by shallow aquifer sands during infiltration, a small fraction can reach the depth of aquifers tapped by shallow wells. In areas where sandy aquifers extend almost to the surface, groundwater dating indicates that recharge is rapid and microbial pathogens are therefore more likely to be present that in areas where sandy aquifers protected by a layer of fine-grained silts and clays and groundwater is older [34]. For reasons that are not fully understood, younger shallow groundwater is typically lower in As than older shallow groundwater. This is the mechanism whereby the local geology appears to control the inverse relationship between As concentrations in shallow tubewells and *E. coli*, and therefore plausibly also the presence of actual pathogens [24], [25]. An alternative explanation for the inverse relationship between childhood diarrhea and As content in groundwater is that As may inactivate pathogens due to its toxicity. This seems unlikely, however, because recent incubations have shown that *E. coli* tolerates As concentrations orders of magnitude higher than As levels in groundwater [25].

Comparing the relative risk of the resulting increase in diarrheal disease to prolonged exposure to As is beyond the scope of this study [22], [23], but the outcome of such an analysis could be that switching to a low-As well, even if it is shallow, remains the preferred course of action. Potential contamination of groundwater from any depth during storage in the home should be taken into account [9], although this particular confounding factor evidently did not overwhelm the relationship between diarrheal disease and As, a characteristic of shallow tubewells that this study shows can now be plausibly linked to the microbial quality of groundwater.

The average daily prevalence of 25 episodes of diarrheal disease per 1000 children under five recorded over twelve 24 hour periods translates into 0.75 episodes per child per month and 3 episodes per *bari* per month based on the average number of 4 children per *bari* (Table 1). If As can indeed be used as proxy for the microbial quality of groundwater, our analysis shows that the microbial quality of groundwater could be a significant factor contributing to the continuing high prevalence of diarrheal disease in Bangladesh and other countries with high population density, poor sanitation, and similar aquifer geology.

The lack of a relationship between childhood diarrhea and As for intermediate wells suggests that, unlike shallow wells, there may not be a pathway of fecal contamination that leads from latrines and ponds to intermediate-depth wells (Table 4). At the same time, a separate analysis of the same Matlab data that considers population density and well depth only has shown that intermediate-depth wells are on average associated with significantly higher levels of childhood diarrhea than either shallow wells or deep wells, regardless of their As content (Table S3). The reason for this association is presently unknown.

Our study has several limitations. First, for a significant proportion of *baris*, assignment of a specific tubewell was based on Euclidian distance between the centroid of the area covered by the *bari* and the location of the nearest tubewell. In some cases, the closest tubewell may not be the one people charged with collecting water go to if that well is owned by a neighboring *bari*. To address this problem, we examined the 2800 *baris* for which a tubewell was unambiguously assigned using the same multivariate logistic regression analysis. The results show again a significant inverse association between childhood diarrhea and As content in shallow wells (OR = 0.88, p = 0.023), but not in intermediate-depth wells (OR = 1.03, p = 0.438) (Table S4). A significant proportion of households probably also switched their source of drinking water after their well was tested for As and deep low-As community wells were installed, and this could have affected diarrheal disease patterns. However, the inverse association between childhood diarrhea and As content in groundwater holds before (2000-2002) and after (2004-2006) the 2003 As testing campaign (Table S5).

In spite of these limitations, we believe that the inverse relationship between the As content of shallow wells and the likelihood of diarrheal disease is an important finding and should be considered in future planning efforts to reduce As exposure. Further study is clearly required, but the potential policy implications of this study may be that, in Matlab at least, households should be discouraged from switching to a low-As well in the intermediate-depth range if deep low-As well is available within walking distance. If no deep well is available, then a shallow well that is low in As may be preferable over an intermediate-depth well that is low in As. In all cases, the As content of groundwater should be monitored periodically given the possibility of intrusion of high-As groundwater from elsewhere [35], [36]. There is no obvious reason why these recommendations should not apply to other fluvio-deltaic systems throughout South and Southeast Asia where the population density is high, sanitation infrastructure is limited, and elevated groundwater As levels have been reported.

## Supporting Information

Figure S1 **The tubewell number and average As content in groundwater at each depth interval (depth interval = 10feet)**
(TIF)

Table S1 **Associations between childhood diarrhea and tubewell As derived by univariate logistic regressions**. As concentrations were classified into 3 levels: 1: very low As (≤10 µg/L), 2: low As (10-50 µg/L) and 3: high As(>50 µg/L). The very low As group was taken as the baseline group in the comparison. Diarrhea data were divided into two datasets: watery diarrhea and bloody diarrhea. Each dataset was analyzed using univariate logistic regression to examine the relationship between childhood diarrhea and tubewell arsenic. Bangladesh is commonly recognized in 3 seasons: a hot, muggy summer from March to June; a rainy monsoon season from June/July to October/November; and a dry winter from November/December to February. According to this, we stratified the diarrhea data into 3 seasons: hot summer from March to June, rainy monsoon season from July to October and dry winter from November to February. Since our analysis was based on the *bari*-level, not on the individual level, it cannot stratify the analysis by age of children directly. Therefore, we selected *baris* having children at the same age to conduct the analysis and *baris* having children with mixed ages were excluded from the analysis.
(DOCX)

Table S2 **Multivariate analysis of associations between childhood diarrhea and arsenic, density of children, household wealth, and tubewell density.** Density of children was calculated by counting the total number of children around a *bari* within 100 meters divided by the area. The tubewell density was calculated in the same way and then categorized into 3 groups which are roughly equal in size.
(DOCX)

Table S3 **Associations of childhood diarrhea with tubewell depth after for adjusting flood control, population density, and socioeconomic status.** The results from this table indicated that children drinking water from intermediate-depth wells (140–300 ft) had a significantly higher risk of diarrheal diseases than those drinking water from shallow wells (10–140ft), while children drinking water from deep wells (≥300 ft) had a lower risk of diarrheal diseases than those drinking water from shallow wells (10–140ft), but the difference was not statistically significant.
(DOCX)

Table S4 **Associations between childhood diarrhea and tubewell arsenic, population density, wealth index and flood control for** ***baris*** **which a tubewell was unambiguously assigned in 142 villages.**
(DOCX)

Table S5 **The association between childhood diarrhea and As in different time periods after adjusting for flood control, population density and socioeconomic status.**
(DOCX)
