# Correction: Increase in Diarrheal Disease Associated with Arsenic Mitigation in Bangladesh

**DOI:** 10.1371/annotation/75d95a55-a58d-4831-8de4-d0f935fe512d

**Published:** 2012-01-27

**Authors:** Jianyong Wu, Alexander van Geen, Kazi Matin Ahmed, Yasuyuki Akita, Janahgir Alam, Patricia J. Culligan, Veronica Escamilla, John Feighery, Andrew S. Ferguson, Peter Knappett, Brian J. Mailloux, Larry D. McKay, Marc L. Serre, P. Kim Streatfield, Mohammad Yunus, Michael Emch

There is a formatting error in the author byline. "Yasuyuki Akita Jahangir Alam ^1,3^" is incorrect. The correct author names and affiliations are: "Yasuyuki Akita^1^, Jahangir Alam^3^”.

