# Supplementary figures and images for: Increase in Diarrheal Disease Associated with Arsenic Mitigation in Bangladesh
Source: PLoS One. 2011 Dec 28;6(12):e29593. doi: 10.1371/journal.pone.0029593 (PMC3247276; doi:10.1371/journal.pone.0029593)

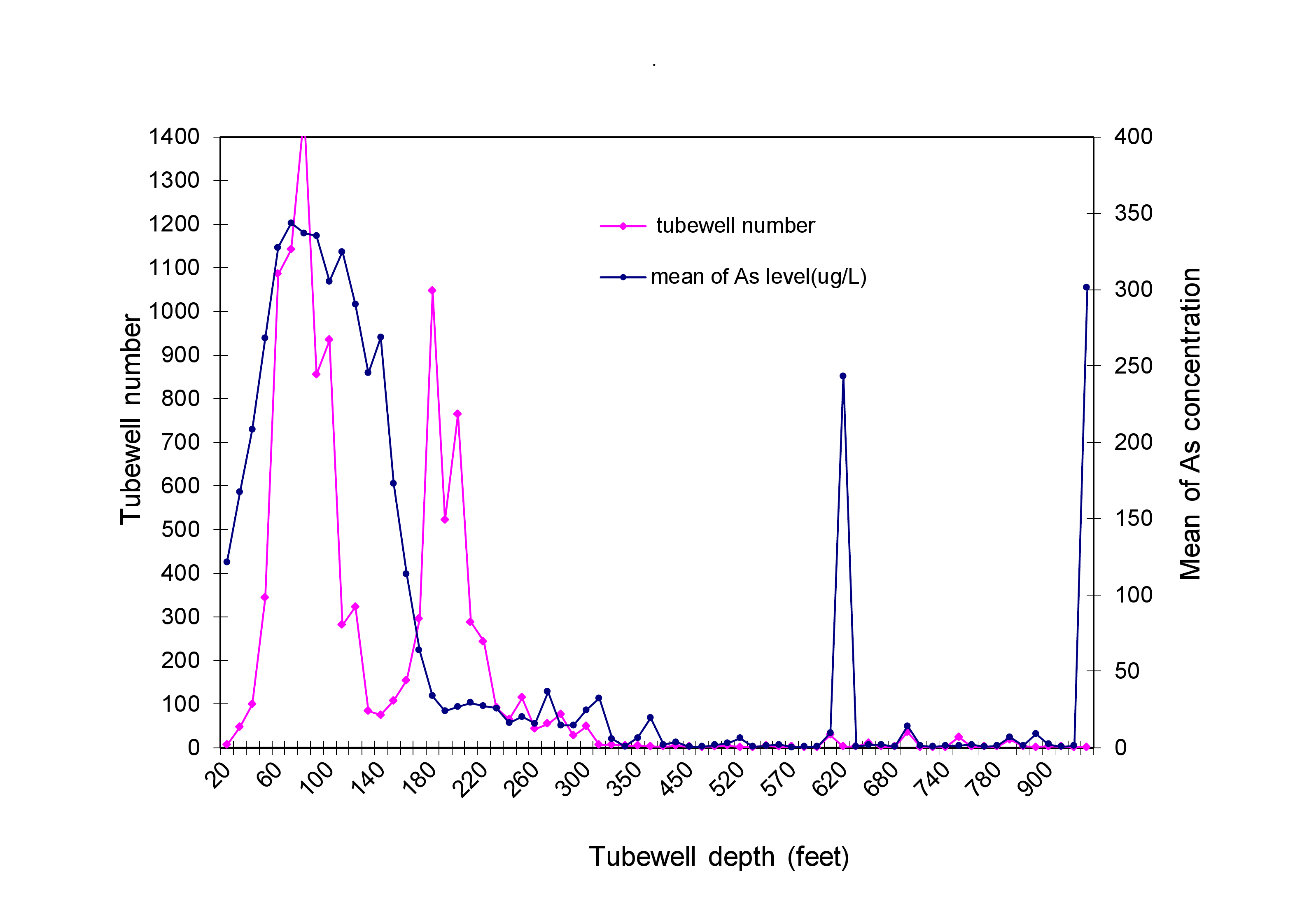

Supplement: Figure S1 — The tubewell number and average As content in groundwater at each depth interval (depth interval = 10feet) (TIF) [file pone.0029593.s001.tif]
