# Supplementary material for: Increase in Diarrheal Disease Associated with Arsenic Mitigation in Bangladesh
Source: PLoS One. 2011 Dec 28;6(12):e29593. doi: 10.1371/journal.pone.0029593 (PMC3247276; doi:10.1371/journal.pone.0029593)
Supplement: Table S1 — Associations between childhood diarrhea and tubewell As derived by univariate logistic regressions. As concentrations were classified into 3 levels: 1: very low As (≤10 µg/L), 2: low As (10-50 µg/L) and 3: high As(>50 µg/L). The very low As group was taken as the baseline group in the comparison. Diarrhea data were divided into two datasets: watery diarrhea and bloody diarrhea. Each dataset was analyzed using univariate logistic regression to examine the relationship between childhood diarrhea and tubewell arsenic. Bangladesh is commonly recognized in 3 seasons: a hot, muggy summer from March to June; a rainy monsoon season from June/July to October/November; and a dry winter from November/December to February. According to this, we stratified the diarrhea data into 3 seasons: hot summer from March to June, rainy monsoon season from July to October and dry winter from November to February. Since our analysis was based on the bari-level, not on the individual level, it cannot stratify the analysis by age of children directly. Therefore, we selected baris having children at the same age to conduct the analysis and baris having children with mixed ages were excluded from the analysis. (DOCX) [file pone.0029593.s002.docx]

| Control variables | n | p | OR | 95%CI |
| --- | --- | --- | --- | --- |
| Diarrhea type |  |  |  |  |
| Watery | 50162 | <0.001 | 0.93 | 0.91-0.95 |
| Bloody | 50162 | 0.674 | 1.01 | 0.97-1.04 |
| Season |  |  |  |  |
| Hot summer | 22913 | 0.002 | 0.95 | 0.93-0.98 |
| Rainy monsoon | 22913 | 0.014 | 0.94 | 0.94-0.99 |
| Dry winter | 22913 | 0.009 | 0.96 | 0.93-0.99 |
| Children age |  |  |  |  |
| 0-12 months | 2887 | 0.613 | 0.97 | 0.86-1.09 |
| 13-24 months | 3516 | 0.079 | 0.93 | 0.86-1.01 |
| 25-36 months | 3927 | 0.006 | 0.90 | 0.83-0.97 |
| 37-48 months | 3800 | 0.025 | 0.91 | 0.83-0.99 |
| 49-50 months | 3287 | 0.048 | 0.90 | 0.90-1.00 |
