# Supplementary material for: Increase in Diarrheal Disease Associated with Arsenic Mitigation in Bangladesh
Source: PLoS One. 2011 Dec 28;6(12):e29593. doi: 10.1371/journal.pone.0029593 (PMC3247276; doi:10.1371/journal.pone.0029593)
Supplement: Table S2 — Multivariate analysis of associations between childhood diarrhea and arsenic, density of children, household wealth, and tubewell density. Density of children was calculated by counting the total number of children around a bari within 100 meters divided by the area. The tubewell density was calculated in the same way and then categorized into 3 groups which are roughly equal in size. (DOCX) [file pone.0029593.s003.docx]

| Control variables | Independent variables | n | p | OR | 95%CI |
| --- | --- | --- | --- | --- | --- |
| Unstratified | As | 49475 | <0.001 | 0.91 | 0.89-0.93 |
|  | Density of children |  | <0.001 | 1.18 | 1.15-1.21 |
|  | Flood control |  | <0.001 | 0.89 | 0.87-0.91 |
|  | Wealth index |  | <0.001 | 0.89 | 0.87-0.91 |
|  | Tubewell density |  | <0.001 | 0.84 | 0.81-0.88 |
| Shallow wells | As | 28654 | 0.001 | 0.89 | 0.83-0.95 |
|  | Density of children |  | <0.001 | 1.02 | 1.01-1.02 |
|  | Flood control |  | <0.001 | 0.79 | 0.74-0.83 |
|  | Wealth index |  | <0.001 | 0.90 | 0.88-0.93 |
|  | Tubewell density |  | <0.001 | 0.89 | 0.86-0.92 |
| Intermediate- depth wells | As | 16321 | 0.223 | 0.98 | 0.94-1.02 |
|  | Density of children |  | <0.001 | 1.02 | 1.02-1.03 |
|  | Flood control |  | 0.041 | 0.93 | 0.87-1.00 |
|  | Wealth index |  | <0.001 | 0.89 | 0.86-0.92 |
|  | Tubewell density |  | <0.001 | 0.90 | 0.86-0.94 |
