# Supplementary material for: Increase in Diarrheal Disease Associated with Arsenic Mitigation in Bangladesh
Source: PLoS One. 2011 Dec 28;6(12):e29593. doi: 10.1371/journal.pone.0029593 (PMC3247276; doi:10.1371/journal.pone.0029593)
Supplement: Table S3 — Associations of childhood diarrhea with tubewell depth after for adjusting flood control, population density, and socioeconomic status. The results from this table indicated that children drinking water from intermediate-depth wells (140–300 ft) had a significantly higher risk of diarrheal diseases than those drinking water from shallow wells (10–140ft), while children drinking water from deep wells (≥300 ft) had a lower risk of diarrheal diseases than those drinking water from shallow wells (10–140ft), but the difference was not statistically significant. (DOCX) [file pone.0029593.s004.docx]

| Depth comparison | n | OR | 95% CI of OR | | p |
| --- | --- | --- | --- | --- | --- |
| Intermediate- depth wells vs. shallow wells | 44975 | 1.26 | 1.21 | 1.31 | <0.001 |
| Deep wells vs. shallow wells | 29425 | 0.90 | 0.77 | 1.06 | 0.202 |
