# Supplementary material for: Increase in Diarrheal Disease Associated with Arsenic Mitigation in Bangladesh
Source: PLoS One. 2011 Dec 28;6(12):e29593. doi: 10.1371/journal.pone.0029593 (PMC3247276; doi:10.1371/journal.pone.0029593)
Supplement: Table S4 — Associations between childhood diarrhea and tubewell arsenic, population density, wealth index and flood control for baris which a tubewell was unambiguously assigned in 142 villages. (DOCX) [file pone.0029593.s005.docx]

| Control variables | Independent variables | *Baris* with matched wells in 142 villages | | | |
| --- | --- | --- | --- | --- | --- |
|  |  | n | p | OR | 95%CI |
| Shallow wells | As | 11617 | 0.023 | 0.88 | 0.78-0.98 |
|  | Flood control |  | <0.001 | 0.67 | 0.61-0.73 |
|  | Population density |  | 0.001 | 1.08 | 1.03-1.14 |
|  | wealth index |  | <0.001 | 0.88 | 0.84-0.92 |
| Intermediate- depth wells | As | 5651 | 0.438 | 1.03 | 0.96-1.11 |
|  | Flood control |  | 0.506 | 0.96 | 0.86-1.08 |
|  | Population density |  | 0.004 | 1.10 | 1.03-1.18 |
|  | wealth index |  | <0.001 | 0.85 | 0.80-0.90 |
