# Supplementary material for: Increase in Diarrheal Disease Associated with Arsenic Mitigation in Bangladesh
Source: PLoS One. 2011 Dec 28;6(12):e29593. doi: 10.1371/journal.pone.0029593 (PMC3247276; doi:10.1371/journal.pone.0029593)
Supplement: Table S5 — The association between childhood diarrhea and As in different time periods after adjusting for flood control, population density and socioeconomic status. (DOCX) [file pone.0029593.s006.docx]

| Time period | Depth | n | p | OR | | 95% CI of OR |
| --- | --- | --- | --- | --- | --- | --- |
| 2000-2002 | Shallow wells | 12027 | 0.017 | | 0.88 | 0.90-0.98 |
|  | Intermediate- depth wells | 6848 | 0.259 | | 0.97 | 0.91-1.03 |
| 2004-2006 | Shallow wells | 12474 | 0.014 | | 0.88 | 0.79-097 |
|  | Intermediate- depth wells | 5651 | 0.496 | | 1.02 | 0.96-1.09 |
